# Global research trends and emerging themes in amino acid deprivation based cancer therapy: a bibliometric and visualization study

**DOI:** 10.3389/fimmu.2026.1832741

**Published:** 2026-06-03

**Authors:** Xiuli He, Tao Cai, Guoqin Zhang, Shulin Cheng, Wensheng Yue

**Affiliations:** 1 Department of Ultrasonography, The Affiliated Hospital of North Sichuan Medical College, Nanchong, Sichuan, China; 2 Department of Urology, The Affiliated Hospital of North Sichuan Medical College, Nanchong, Sichuan, China; 3 Dazhou Dachuan District People’s Hospital (Dazhou Third People’s Hospital), Dazhou, Sichuan, China

**Keywords:** amino acid deprivation, arginine deprivation, bibliometric analysis, cancer metabolism, methionine restriction

## Abstract

**Background:**

Amino acid deprivation and restriction have emerged as metabolically informed anticancer strategies, encompassing dietary interventions, enzyme-mediated depletion, and pathway-targeted approaches. The fast-growing and highly interdisciplinary literature makes it difficult to obtain an integrated view of research foundations and evolving hotspots.

**Methods:**

Publications on amino acid deprivation, restriction, starvation, or depletion in cancer (2006–2025) were retrieved from the Web of Science Core Collection and cross-validated using PubMed. Bibliometric and visualization analyses were conducted with Microsoft Excel 2021, VOSviewer, CiteSpace, Scimago Graphica, and Charticulator to map publication trends, leading contributors, collaboration networks, journal landscapes, keyword co-occurrence/clustering, and reference co-citation and burst patterns.

**Results:**

The literature showed sustained growth over 2006-2025, with expanding global participation. Collaboration analyses identified major contributing countries, institutions, and author groups, with a network structure characterized by highly connected hubs. Keyword mapping indicated that research themes are anchored in tumor metabolic reprogramming and amino acid-centered vulnerabilities, and have progressively diversified toward regulated cell-death programs and clinically oriented strategies, including enzyme-based depletion approaches and combination regimens. Co-citation and burst analyses highlighted influential conceptual frameworks in cancer metabolism and landmark translational studies that shaped subsequent research directions.

**Conclusions:**

This bibliometric study delineates the knowledge structure and thematic evolution of amino acid deprivation-related oncology research from 2006 to 2025. The findings provide a quantitative reference to facilitate rapid orientation, identify influential contributions, and support future mechanistic investigations and translational development.

## 1 Introduction

Cancer remains a leading cause of morbidity and mortality worldwide, and therapeutic resistance continues to limit durable benefits from surgery, chemotherapy, radiotherapy, targeted agents, and immunotherapy (1, 2). A growing body of evidence indicates that metabolic adaptation is a central hallmark of malignant progression and treatment failure, enabling tumor cells to survive in nutrient-poor microenvironments and to withstand therapeutic stress (3, 4). Among metabolic dependencies, amino acid utilization has attracted particular attention because amino acids are not only substrates for protein synthesis, but also essential inputs for redox control, nucleotide biosynthesis, epigenetic regulation, and signaling pathways that govern cell growth and survival (5, 6). Consequently, strategies that restrict, deplete, or pharmacologically disrupt amino acid availability have emerged as an actionable approach to expose metabolic vulnerabilities in cancer.

Amino acid deprivation–based interventions span multiple modalities and biological contexts (7). Dietary restriction paradigms, including methionine restriction, have been explored for their capacity to rewire systemic metabolism and sensitize tumors to standard therapies (8, 9). Enzyme-mediated depletion approaches, such as L-asparaginase for asparagine depletion and arginine-depleting enzymes for arginine auxotrophy, provide clinically tractable tools to target tumor-specific metabolic liabilities (10). Mechanistically, amino acid perturbation intersects with key cellular programs including oxidative stress responses, nutrient-sensing signaling such as mTOR, and regulated cell death pathways including apoptosis and autophagy, while newly recognized modes of cell death have further expanded the conceptual space of amino acid–linked cancer vulnerabilities (11, 12). At the translational level, increasing attention has been directed toward combination regimens that integrate amino acid targeting with chemotherapy, radiotherapy, and immunomodulatory approaches, as well as toward the challenges of metabolic plasticity and resistance (13, 14).

Bibliometric analysis offers a complementary, systematic way to quantify scientific output, map collaboration and knowledge networks, and track thematic evolution at scale (15). By integrating large bibliographic datasets, bibliometric approaches can identify influential journals and references, reveal collaboration patterns across countries, institutions, and authors, and detect emerging topics through keyword dynamics and citation bursts. Accordingly, a comprehensive bibliometric and visualization analysis of amino acid deprivation–related cancer research may facilitate rapid orientation for researchers and clinicians, highlight translational opportunities, and inform future study design.

In this study, publications on amino acid deprivation, restriction, starvation, or depletion in cancer were retrieved from major bibliographic databases over the period 2006–2025. A series of bibliometric and visualization analyses were conducted to characterize publication trends, core contributors, collaboration structures, and thematic evolution, with additional cross-database comparison used to strengthen robustness and interpretability. The resulting overview is intended to provide a panoramic reference for the field and to support future mechanistic and translational advances in amino acid–targeted oncology.

## 2 Materials and methods

### 2.1 Data acquisition

A literature search was performed in the Web of Science Core Collection (WoSCC) using the following query: TS = (“Neoplasms” OR “Tumors” OR “Neoplasia” OR “Neoplasias” OR “Neoplasm” OR “Tumor” OR “Cancer” OR “Cancers” OR “Malignancy” OR “Malignancies”) AND TS = (“Amino Acid” OR “Glutamine” OR “Arginine” OR “Methionine” OR “Glutamine” OR “Cystine” OR “Cysteine” OR “Asparagine” OR “Serine” OR “Glycine”) AND TS = (“Deprivation” OR “Starvation” OR “Restriction” OR “Depletion”). This search retrieved 5,563 records published between 2006 and 2025. Only English-language articles and reviews were eligible. A total of 273 non-eligible records were excluded, including News Item (n=1), Correction (n=2), Letter (n=6), Retracted Publication (n=8), Book Chapters (n=25), Early Access (n=30), Proceeding Paper (n=45), Editorial Material (n=59), Meeting Abstract (n=78), and Non-English publications (n=19). Ultimately, 5,290 records were retained, comprising 4,557 articles and 733 reviews.

**Figure 1:**
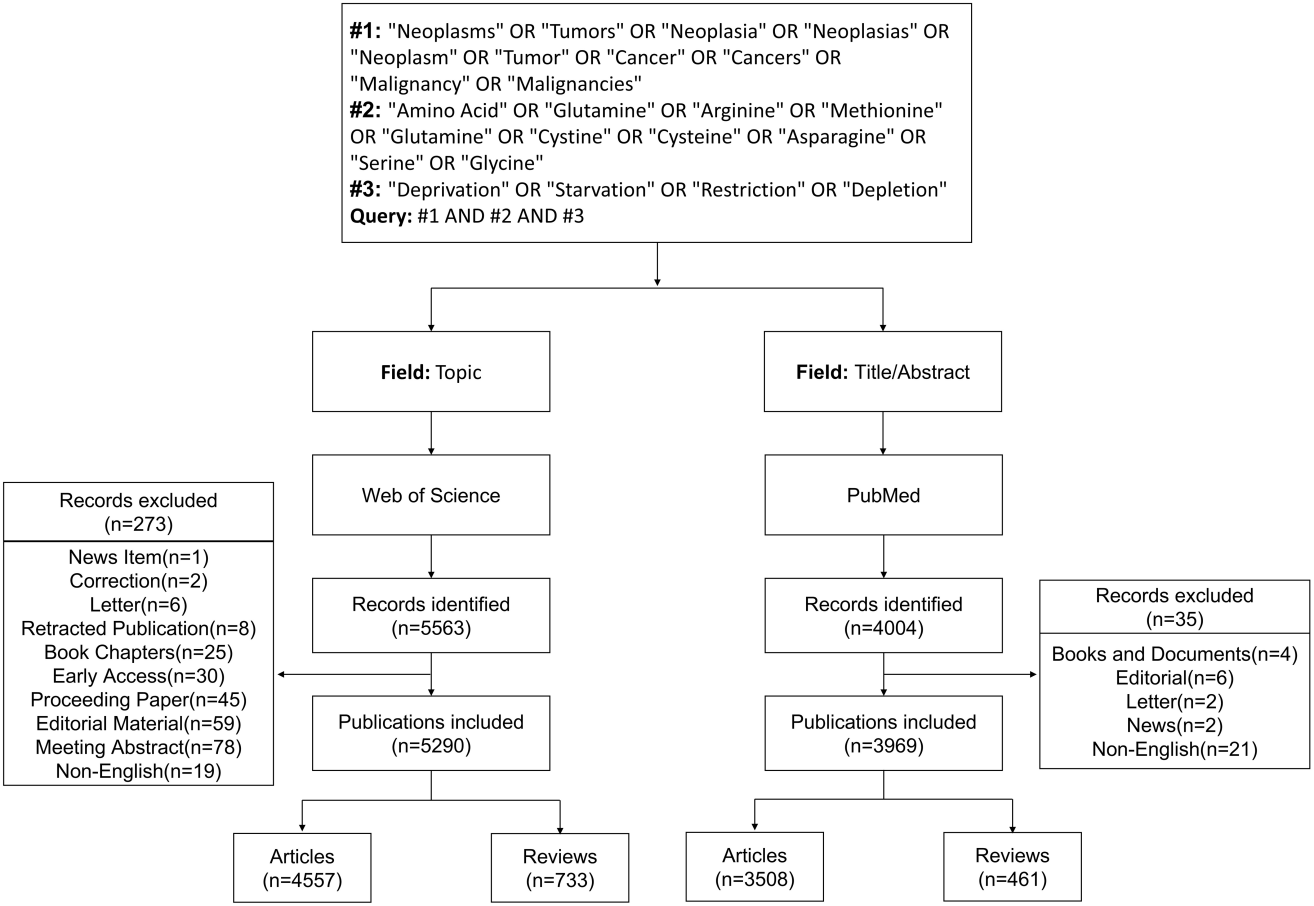
Flowchart of data retrieval and processing. Search strategies in WoSCC and PubMed, inclusion/exclusion criteria, record screening, and final datasets retained for bibliometric analyses.

In parallel, PubMed was queried using: (Neoplasms[Title/Abstract] OR Tumors[Title/Abstract] OR Neoplasia[Title/Abstract] OR Neoplasias[Title/Abstract] OR Neoplasm[Title/Abstract] OR Tumor[Title/Abstract] OR Cancer[Title/Abstract] OR Cancers[Title/Abstract] OR Malignancy[Title/Abstract] OR Malignancies[Title/Abstract]) AND (Amino Acid[Title/Abstract] OR Glutamine[Title/Abstract] OR Arginine[Title/Abstract] OR Methionine[Title/Abstract] OR Glutamine[Title/Abstract] OR Cystine[Title/Abstract] OR Cysteine[Title/Abstract] OR Asparagine[Title/Abstract] OR Serine[Title/Abstract] OR Glycine[Title/Abstract]) AND (Deprivation[Title/Abstract] OR Starvation[Title/Abstract] OR Restriction[Title/Abstract] OR Depletion[Title/Abstract]). A total of 4,004 records published during 2006–2025 were identified. The inclusion criteria were restricted to English-language articles and reviews, and 35 non-eligible records were removed, including Books and Documents (n=4), Editorial (n=6), Letter (n=2), News (n=2), and Non-English publications (n=21). The final PubMed dataset comprised 3,969 records, including 3,508 articles and 461 reviews. The detailed workflow of data retrieval and screening is illustrated in **Figure 1**.

### 2.2 Bibliometric analysis and visualization

Following data curation, bibliometric analyses and visualizations were conducted using Microsoft Excel 2021, VOSviewer, CiteSpace, Charticulator, and Scimago Graphica. Microsoft Excel 2021 was used to summarize annual publication outputs in the Web of Science dataset (2006–2025) to depict overall growth trends. VOSviewer (v1.6.20) was applied to map and visualize bibliometric networks, including country-level collaboration, author and institutional cooperation, and relationships among productive and highly cited journals. VOSviewer (Visualization of Similarities Viewer), developed by Nees Jan van Eck and Ludo Waltman, is a widely used tool for constructing and displaying bibliometric networks (16).

To better illustrate temporal patterns, Scimago Graphica was used to present year-by-year publication trajectories for the most productive countries. In addition, Charticulator was employed to generate chord diagrams for international collaboration; Charticulator is a Microsoft Research project that enables code-free, web-based customization of data visualizations. Finally, CiteSpace (v6.4.R1) was utilized for dual-map overlay of journals, keyword co-occurrence analysis, reference co-citation analysis, and burst detection for both keywords and references, with the resulting networks visualized accordingly. CiteSpace is a bibliometric visualization platform developed by Professor Chaomei Chen and colleagues (17). The time span was set to 2006–2025 with 1-year per slice. For the keyword analysis, terms were first extracted from the 5,290 WoSCC records using the g-index with k = 8, yielding 304 keywords. A thesaurus-based cleaning step was then conducted: synonymous terms were merged, whereas non-informative or irrelevant terms were removed. After generating the merge/delete file, the analysis was rerun. To minimize the emergence of newly introduced keywords, k was reduced to 3 prior to re-analysis, resulting in 162 finalized keywords, which were subsequently visualized. For the PubMed dataset (3,969 records), keyword extraction was performed in the same manner. Using the g-index with k = 10, 258 keywords were obtained. After synonym consolidation and removal of uninformative terms, the analysis was rerun with k lowered to 5, producing 165 keywords for visualization. The PubMed-derived keyword results were used to validate and complement the findings from the WoSCC-based analysis.

## 3 Results

### 3.1 Global trend in publication outputs and citations

**Figure 2:**
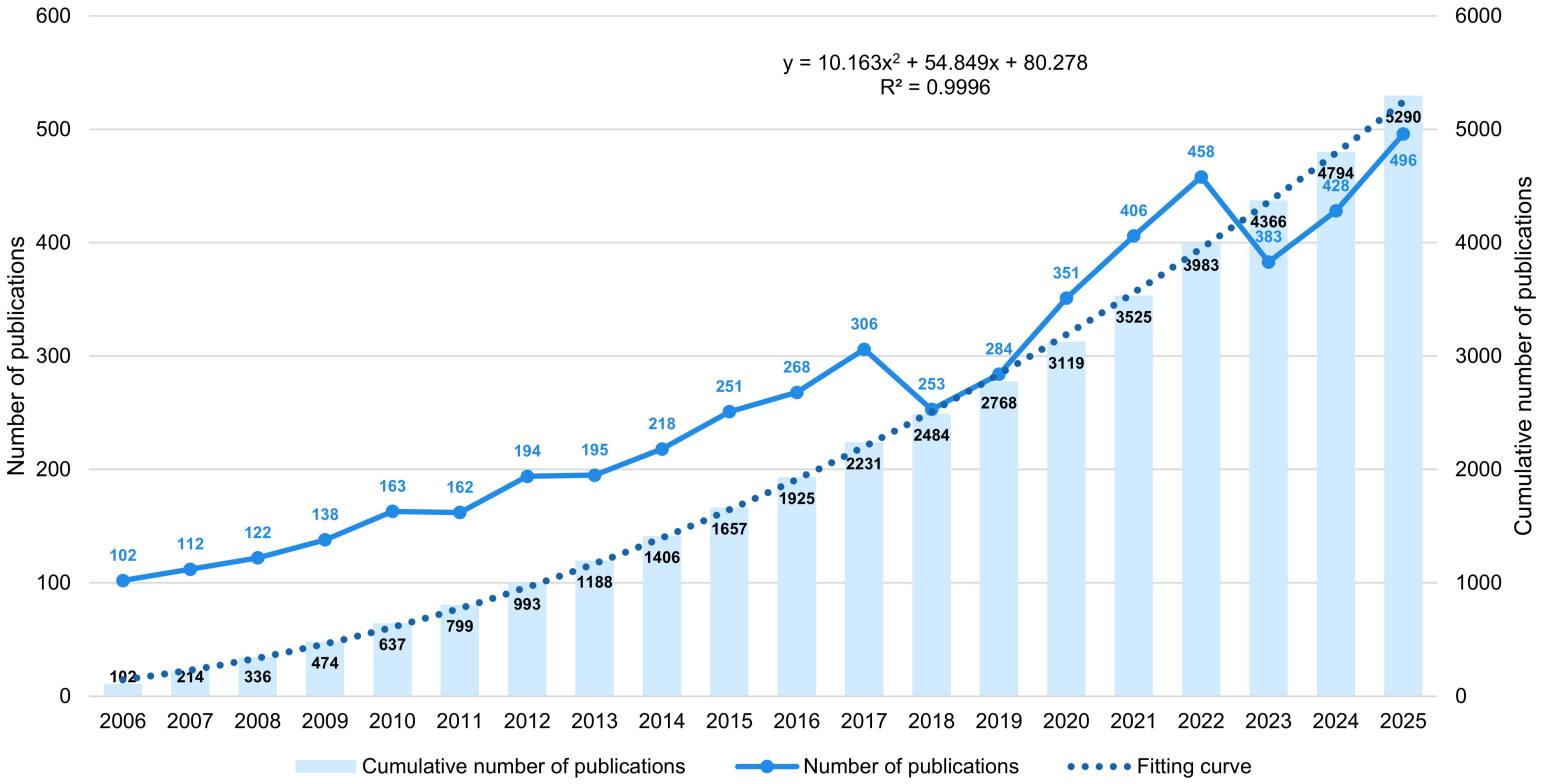
Annual publication trends in amino acid deprivation–related cancer research (2006–2025). Yearly outputs and cumulative publications illustrating the growth trajectory of the field.

**Figure 2** illustrates the annual publication output on amino acid deprivation–based therapy in oncology from 2006 to 2025. The number of publications increased steadily from 102 in 2006 to 496 in 2025, representing an almost fivefold rise, accompanied by a rapid accumulation in the cumulative number of publications, indicating sustained and growing research interest. Although minor, transient dips were observed in 2011 (162 publications), 2018 (253 publications), and 2023 (383 publications), publication output rebounded promptly in subsequent years and entered a pronounced acceleration phase after 2020. Specifically, annual output surpassed 350, 400, and 450 publications in 2020–2022, respectively, and reached its peak at 496 publications in 2025. Collectively, these patterns suggest that tumor metabolism research, particularly amino acid-targeted intervention strategies, has gained increasing academic attention and may remain an active area of investigation. A polynomial model was fitted to the cumulative publication curve (total publications from 2006 to each year), yielding an excellent fit (R² = 0.9996); the fitted trajectory is shown as the blue dashed line in **Figure 2**. This model further supports a persistent upward trend and suggests continued growth in publication output in the near future. Across the 5,290 eligible records, a total of 103 subject categories were involved. **Table 1** presents the top 20 categories by output. Oncology ranked first with 1,438 publications, followed by Biochemistry & Molecular Biology (1,269), Cell Biology (1,157), Multidisciplinary Sciences (415), and Pharmacology & Pharmacy (335).

**Table 1:** Top 20 research categories by number of publications.

Rank	Web of science categories	Record count	Rank	Web of science categories	Record count
1	Oncology	1438	11	Biophysics	134
2	Biochemistry Molecular Biology	1269	12	Nanoscience Nanotechnology	127
3	Cell Biology	1157	13	Materials Science Multidisciplinary	113
4	Multidisciplinary Sciences	415	14	Biotechnology Applied Microbiology	109
5	Pharmacology Pharmacy	335	15	Nutrition Dietetics	102
6	Medicine Research Experimental	268	16	Biology	100
7	Chemistry Multidisciplinary	266	17	Chemistry Medicinal	94
8	Genetics Heredity	228	18	Hematology	91
9	Endocrinology Metabolism	227	19	Toxicology	79
10	Immunology	165	20	Gastroenterology Hepatology	73

### 3.2 Distribution of countries/regions

A total of 86 countries contributed to this research field worldwide. **Table 2** summarizes the top 10 countries by publication output. The United States ranked first in the number of publications (1,983), total citations (161,713), and H-index (185), highlighting its extensive research base and strong academic influence. China ranked second with 1,545 publications, reflecting high research activity; however, its total citations and citations per paper were comparatively lower, and its H-index (102) lagged substantially behind that of the United States, suggesting that overall impact and research quality may still be improved. Traditional research-intensive countries such as Japan, the United Kingdom, and Germany produced fewer publications than the United States and China, yet showed higher citations per paper and relatively strong H-index performance, indicating greater recognition and influence of their outputs.

**Table 2:** Top 10 countries in terms of publication volume.

Rank	Country	NP	NC	AC	H-index
1	USA	1983	161713	81.55	185
2	China	1545	55035	35.62	102
3	Japan	399	15818	39.64	61
4	United Kingdom	384	24900	64.84	82
5	Germany	365	21374	58.56	69
6	South Korea	265	9984	37.68	53
7	Italy	232	11570	49.87	54
8	Canada	174	10127	58.20	54
9	India	163	3500	21.47	33
10	France	161	8432	52.37	50

**Figure 3:**
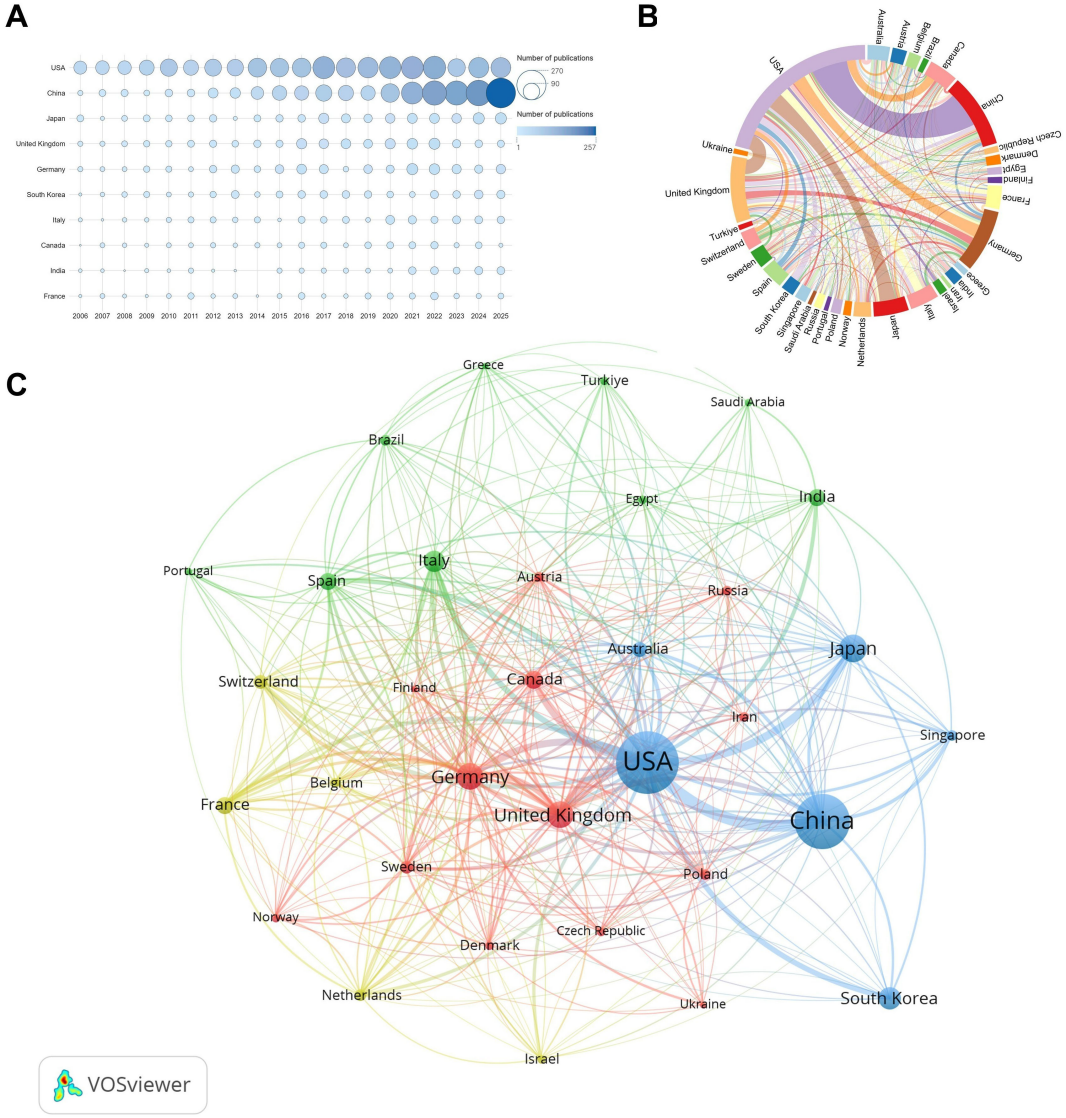
Country-level productivity and international collaboration. **(A)** Annual publication outputs of the top contributing countries. **(B)** Country collaboration network among countries meeting the publication threshold; node size indicates total link strength (TLS) and link thickness indicates collaboration strength. **(C)** Chord diagram visualizing bilateral collaborations between major contributing countries.

**Figure 3A** depicts annual publication trends for the top 10 most productive countries. The United States increased steadily from 50 publications in 2006 to 104 in 2016, peaking at 142 in 2017. Although output declined to 91 in 2023, it remained at a relatively high level overall (reaching 117 in 2025), reflecting sustained productivity. In contrast, China started from a low baseline (8 publications in 2006) with modest early growth, but entered a rapid expansion phase after 2014 (47 publications). Annual output surpassed 100 in 2020 and rose sharply thereafter, reaching 257 publications in 2025-more than twice the U.S. output in the same year-indicating that China has recently become the major driver of annual publication volume in this area. Other leading countries, including Japan, the United Kingdom, and Germany, showed relatively stable annual outputs in the range of approximately 10–40 publications, whereas India exhibited an emerging upward trend after 2021 (17 publications), reaching 19 publications in 2024.

International collaboration was assessed using VOSviewer, including only countries with ≥20 publications (n = 33). To illustrate collaborative patterns, a country collaboration network map and a chord diagram were generated (**Figures 3B, C**). Node size represents Total Link Strength (TLS), defined as the total number of collaborative links a country has with other countries; links between nodes indicate collaboration, and link thickness denotes Link Strength (LS), representing the frequency of co-authorship between two countries. The global collaboration network displayed a highly centralized structure dominated by the United States, which showed the highest TLS (1,160), far exceeding other countries, and maintained extensive collaborations with nearly all major contributors. The strongest bilateral links were observed between the United States and China (LS = 265), Japan (LS = 121), the United Kingdom (LS = 107), and Germany (LS = 94), underscoring the United States as a key hub for global knowledge exchange and cooperation. In parallel, several European countries, particularly the United Kingdom (TLS = 446), Germany (TLS = 420), and France (TLS = 158), formed dense intra-European collaboration clusters while also maintaining strong ties with the United States, collectively constituting a secondary center of the network. Countries such as Japan, South Korea, and Australia appeared to position themselves through close bidirectional collaborations with both the United States and China.

### 3.3 Authors and institutions

A total of 35,615 authors contributed to research in this field. **Table 3** lists the top 10 authors by publication output. Highly productive authors, exemplified by Robert M. Hoffman (NP = 76, NC = 1,577, H = 22) and Han Qinghong (NP = 68, NC = 1,322, H = 21), showed a clear lead in publication volume, reflecting sustained research productivity. In contrast, several high-impact authors, such as John Stephen Bomalaski (NP = 40, NC = 2,069, AC = 51.73, H = 24) and Paul Ning-Man Cheng (NP = 26, NC = 1,271, AC = 48.88, H = 18), were not the most prolific, yet achieved notably higher citation counts and citations per paper. In particular, John Stephen Bomalaski showed the highest H-index among the top authors, indicating broader academic recognition and influence of his work.

**Table 3:** Top 10 authors by number of publications.

Rank	Author	NP	NC	AC	H-index
1	Hoffman, Robert M.	76	1577	20.75	22
2	Han, Qinghong	68	1322	19.44	21
3	Bouvet, Michael	46	730	15.87	16
4	Bomalaski, John Stephen	40	2069	51.73	24
5	Park, Woo Hyun	29	880	30.34	20
6	Mizuta, Kohei	29	184	6.34	8
7	Kang, Byung Mo	28	170	6.07	8
8	Morinaga, Sei	27	204	7.56	8
9	Cheng, Paul Ning-Man	26	1271	48.88	18
10	Igarashi, Kentaro	26	349	13.42	10

**Figure 4:**
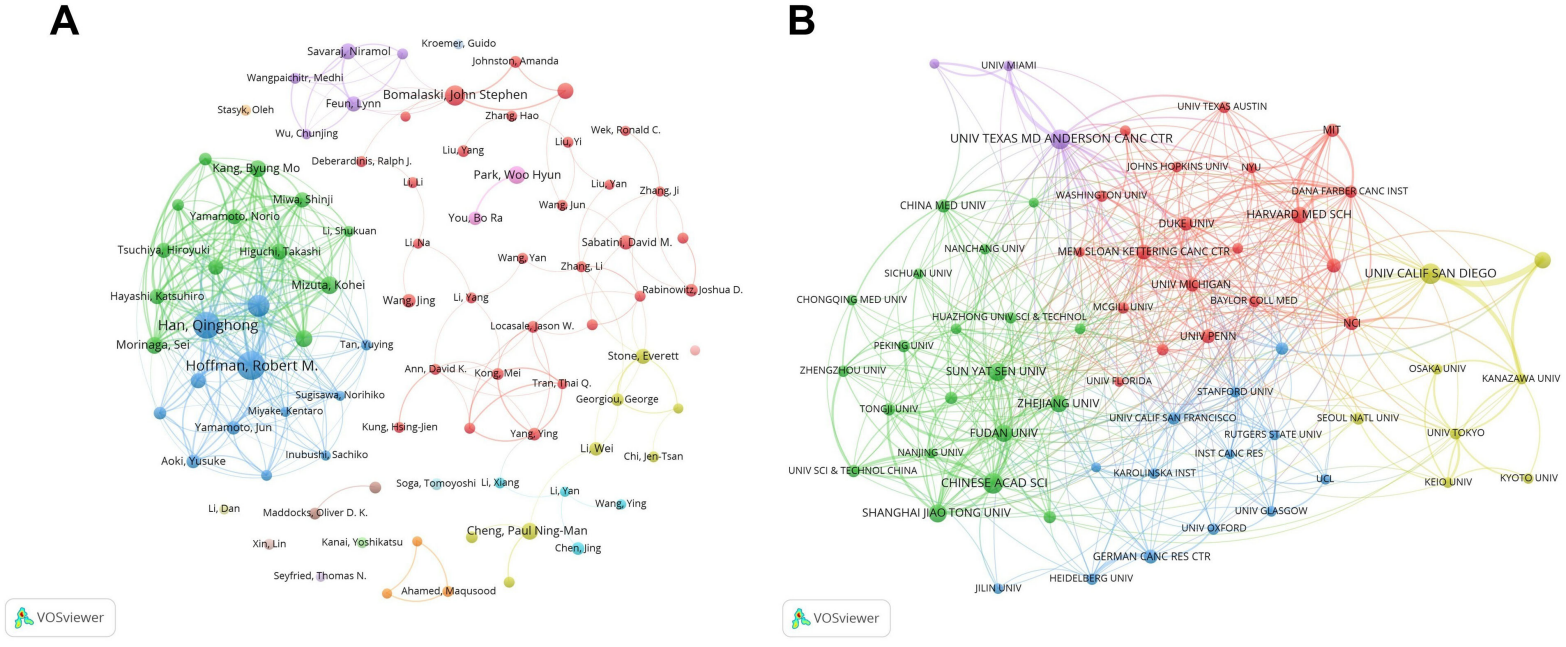
Collaboration networks of authors and institutions. **(A)** Author co-authorship network for authors meeting the minimum publication threshold. **(B)** Institutional collaboration network for institutions meeting the minimum publication threshold.

Author collaboration was further examined using VOSviewer, including authors with ≥10 publications (n = 87). The resulting co-authorship network (**Figure 4A**) revealed a dominant and densely connected collaboration cluster centered on Robert M. Hoffman (TLS = 512) and Han Qinghong (TLS = 495). Beyond their high outputs (NP = 76/68), they maintained strong collaborative ties with numerous co-authors, including Michael Bouvet, Kentaro Igarashi, and Kohei Mizuta; the strongest link was observed between Han Qinghong and Robert M. Hoffman (LS = 67), forming a highly productive core network with intensive internal collaboration. In parallel, another set of influential researchers, represented by David M. Sabatini, Craig B. Thompson, and Joshua D. Rabinowitz, formed secondary clusters that may not have the highest overall link strength but exert substantial influence within specific research directions through key collaborations. Several smaller but tightly knit groups were also identified along with a few authors who met the publication threshold but showed minimal collaboration.

**Table 4** summarizes the top 10 institutions by publication output. Leading U.S. cancer centers—such as The University of Texas MD Anderson Cancer Center (NC = 11,029; AC = 97.60; H = 52) and Memorial Sloan Kettering Cancer Center (NC = 12,715; AC = 208.44), did not necessarily rank first in publication volume (NP = 113/61), yet exhibited markedly higher citations per paper and H-indices, underscoring their strong academic impact. In comparison, major Chinese institutions, including the Chinese Academy of Sciences and top universities (such as Shanghai Jiao Tong University and Sun Yat-sen University), constituted a high-output cluster (NP = 87–114). Notably, the Chinese Academy of Sciences performed strongly in both scale (NP = 114) and influence (NC = 6,208; H = 41). Anticancer Inc., the only industry-based research entity among the top institutions, showed high productivity (NP = 78) but comparatively lower citations per paper (AC = 20.95) and H-index (H = 22), potentially reflecting a stronger emphasis on applied and translational outputs.

**Table 4:** Top 10 institutions by publication volume.

Rank	Author	NP	NC	AC	H-index
1	Hoffman, Robert M.	76	1577	20.75	22
2	Han, Qinghong	68	1322	19.44	21
3	Bouvet, Michael	46	730	15.87	16
4	Bomalaski, John Stephen	40	2069	51.73	24
5	Park, Woo Hyun	29	880	30.34	20
6	Mizuta, Kohei	29	184	6.34	8
7	Kang, Byung Mo	28	170	6.07	8
8	Morinaga, Sei	27	204	7.56	8
9	Cheng, Paul Ning-Man	26	1271	48.88	18
10	Igarashi, Kentaro	26	349	13.42	10

Institutional collaboration was analyzed for organizations with ≥30 publications (n = 64) (**Figure 4B**). MD Anderson Cancer Center (TLS = 105) and the University of California, San Diego (TLS = 100) emerged as the most active collaboration hubs. MD Anderson displayed a broad international network, with strong links to U.S. institutions and frequent collaborations with leading Chinese organizations, forming a prominent trans-Pacific collaboration axis. In addition, a cluster of elite U.S. East Coast institutions, represented by Harvard Medical School, the Massachusetts Institute of Technology, Memorial Sloan Kettering Cancer Center, and Dana-Farber Cancer Institute, formed another dense and historically influential collaboration circle. Notably, some institutions showed relatively high TLS but collaborated with a narrow set of partners, suggesting focused and deep bilateral collaboration patterns.

### 3.4 Journals

**Table 5** (left) summarizes the top 10 journals by publication output. The Journal of Biological Chemistry (USA; IF = 3.9) ranked first with 131 papers, followed by the International Journal of Molecular Sciences (Switzerland; IF = 4.9) and Cancer Research (USA; IF = 16.6) with 113 and 101 papers, respectively. This pattern suggests that these outlets are particularly favored by researchers in this area and have made substantial contributions to the field. Among the top 10 productive journals, Cancer Research had the highest impact factor (IF = 16.6). **Table 5** (right) lists the top 10 most-cited journals. J Biol Chem (Journal of Biological Chemistry; USA; IF = 3.9) received the highest number of citations (11,517), followed by Nature (United Kingdom; IF = 48.5) and Cancer Res (Cancer Research; USA; IF = 16.6) with 9,404 and 9,219 citations, respectively, indicating their strong influence and foundational role within the discipline.

**Table 5:** The top 10 publishing journals by number of publications and the top 10 cited journals by citation frequency.

Rank	Journals	NP	Country	IF (JCR2024)	Cited journals	NC	Country	IF (JCR2024)
1	Journal of biological chemistry	131	USA	3.9	J BIOL CHEM	11517	USA	3.9
2	International journal of molecular sciences	113	Switzerland	4.9	NATURE	9404	United Kingdom	48.5
3	Cancer research	101	USA	16.6	CANCER RES	9219	USA	16.6
4	Plos one	98	USA	2.6	P NATL ACAD SCI USA	8283	USA	9.1
5	Oncogene	92	United Kingdom	7.3	CELL	7935	USA	42.5
6	Oncotarget	90	USA	0	SCIENCE	5986	USA	45.8
7	Scientific reports	79	United Kingdom	3.9	ONCOGENE	4293	United Kingdom	7.3
8	Cancers	76	Switzerland	4.4	MOL CELL	4216	USA	16.6
9	Nature communications	72	United Kingdom	15.7	CELL METAB	4026	USA	30.9
10	Cell death & disease	71	United Kingdom	9.6	NAT COMMUN	3810	United Kingdom	15.7

**Figure 5:**
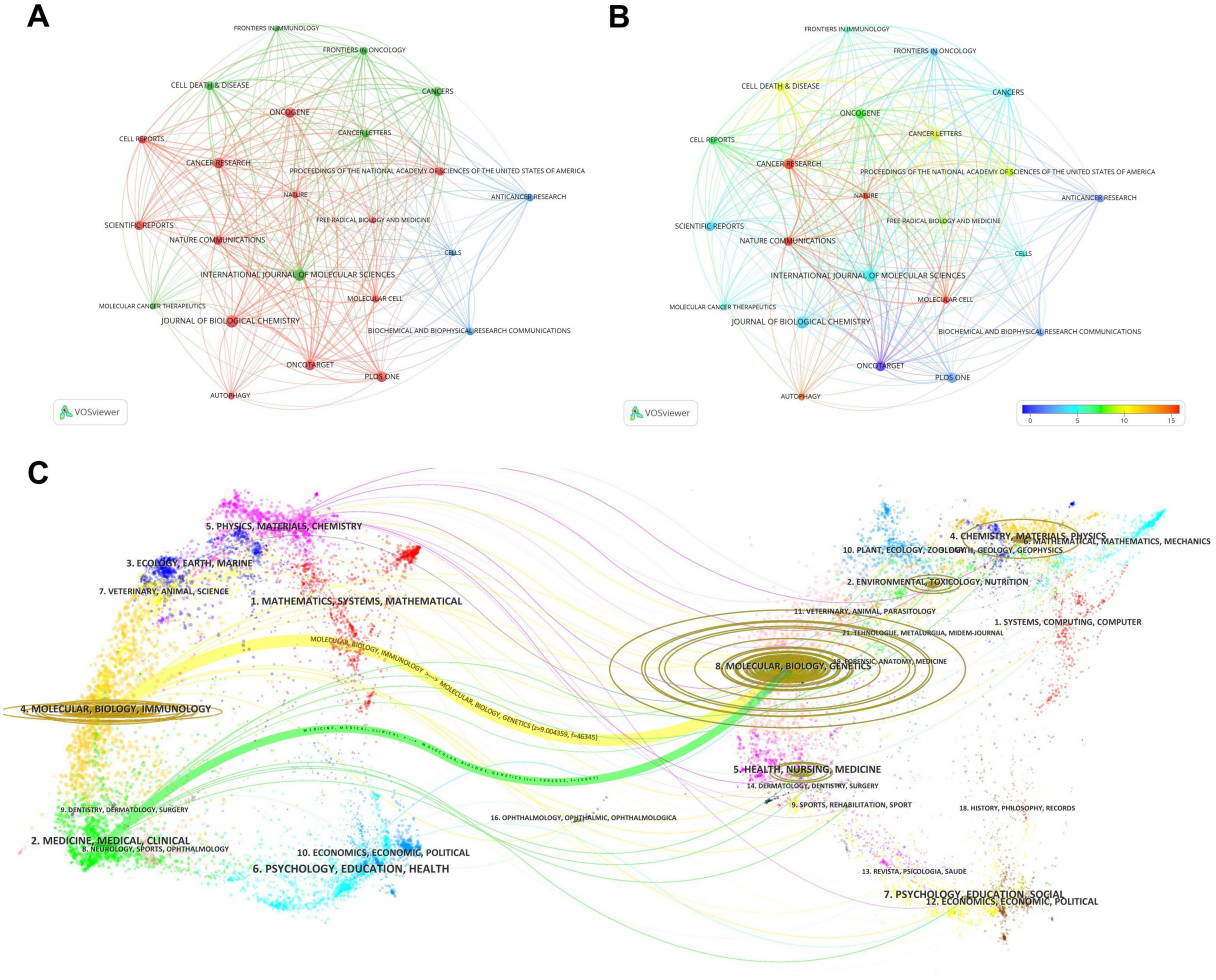
Journal landscape and knowledge flow. **(A)** Journal network based on coupling/co-citation relationships among journals meeting the publication threshold; node size indicates publication output and links represent shared citations. **(B)** Overlay visualization of journal impact factors on the journal network. **(C)** Dual-map overlay of source journals (citing; left) and cited journals (right), with colored trajectories indicating major citation pathways.

**Figure 5A** presents the journal co-citation (coupling) network. A total of 23 journals with ≥30 publications were included; node size corresponds to publication volume, and links indicate that two journals were co-cited by the same article. **Figure 5B** overlays journal impact factors onto the co-citation network, using a color gradient from blue (lower IF) to red (higher IF). **Figure 5C** shows a dual-map overlay of source journals and cited journals for the 5,290 included publications, visualizing cross-domain citation relationships and journal-level knowledge flows between citing fields (left) and cited knowledge bases (right). Clustering based on the built-in Z-score algorithm identified two major citation pathways, encoded by the colors of the cited regions; the width of each trajectory is proportional to the corresponding Z-score. The dominant citation streams originated from “MOLECULAR, BIOLOGY, GENETICS” and extended toward “MOLECULAR, BIOLOGY, IMMUNOLOGY” and “MEDICINE, MEDICAL, CLINICAL.” Notably, the pathway from “MOLECULAR, BIOLOGY, GENETICS” to “MOLECULAR, BIOLOGY, IMMUNOLOGY” exhibited the highest Z value (z = 9.004), underscoring its prominence and impact in shaping knowledge diffusion.

### 3.5 Keywords and hotspots

**Figure 6:**
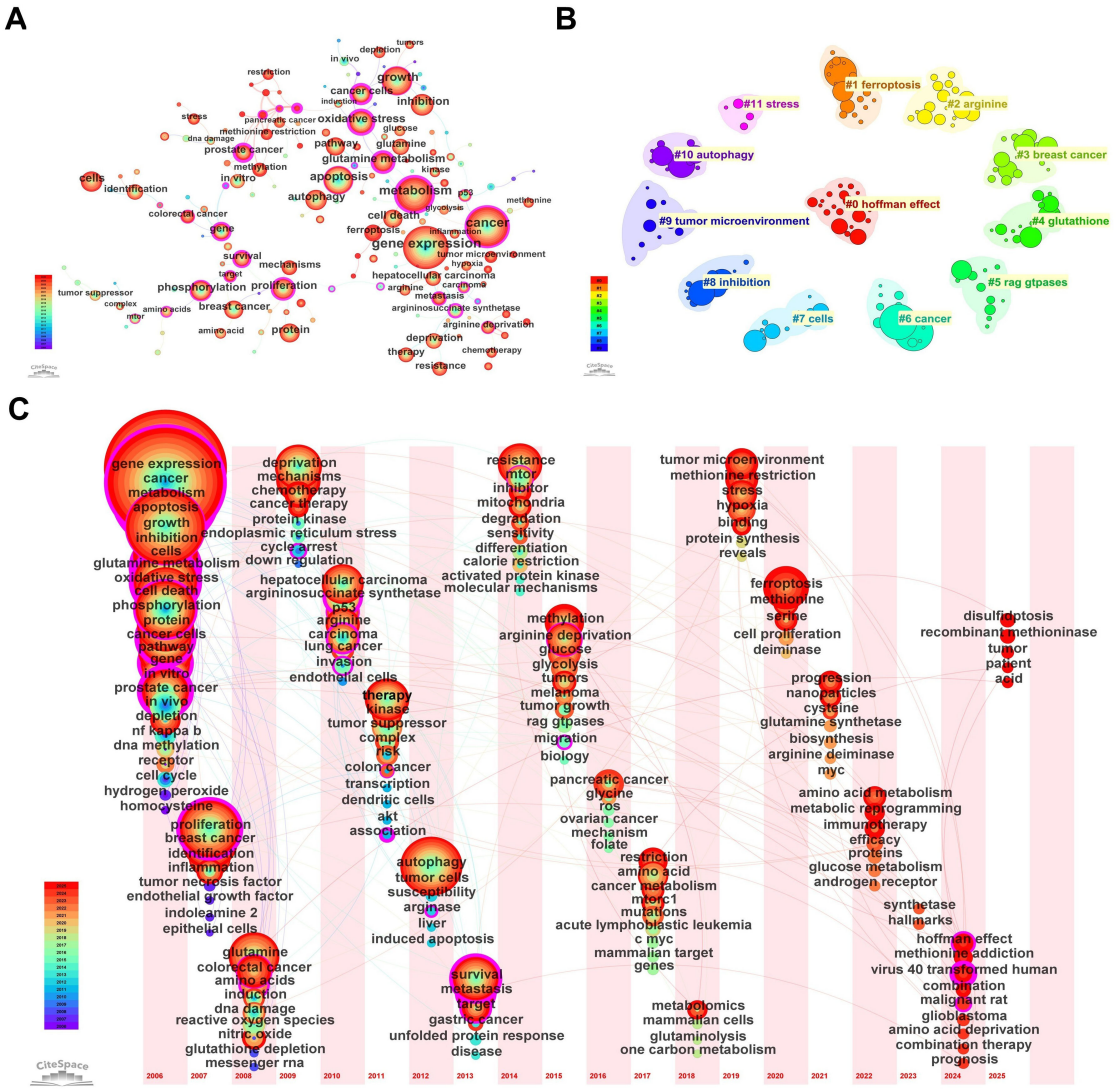
Keyword mapping and thematic evolution (WoSCC). **(A)** Keyword co-occurrence network; node size represents keyword frequency and links indicate co-occurrence relationships. **(B)** Keyword clustering map generated by CiteSpace (LLR algorithm). **(C)** Keyword time-zone visualization showing the temporal distribution and evolution of major themes across 2006–2025.

**Figure 6A** shows the keyword co-occurrence network, in which nodes represent keywords and node size corresponds to keyword frequency (larger nodes indicate higher occurrence). Overall, the intellectual core of this field is strongly centered on metabolic reprogramming, with a particular emphasis on amino acid–related networks dominated by glutamine metabolism (frequency = 408; centrality = 0.77). This theme is closely connected to oxidative stress (frequency = 395; centrality = 0.69), regulated cell death programs (including apoptosis and autophagy), and major signaling processes, together forming the mechanistic backbone of the literature. In addition, highly central terms such as argininosuccinate synthetase (centrality = 0.57) and prostate cancer (centrality = 0.62) suggest that precision interventions targeting tumors with defined metabolic liabilities represent an important translational direction. Notably, ferroptosis has already formed a sizeable cluster (frequency = 165) but shows relatively low centrality (0.07), implying a rapidly expanding yet comparatively independent subdomain. In contrast, keywords related to the tumor microenvironment, immunotherapy, and combination therapy currently exhibit modest centrality, indicating that while these cross-cutting frontiers are gaining attention, their roles as network bridges have not yet been fully established.

Based on the co-occurrence network, keyword clustering was conducted (**Figure 6B**). Cluster labels were extracted from the keyword field using CiteSpace’s log-likelihood ratio (LLR) algorithm, yielding 12 clusters: #0 hoffman effect, #1 ferroptosis, #2 arginine, #3 breast cancer, #4 glutathione, #5 rag gtpases, #6 cancer, #7 cells, #8 inhibition, #9 tumor microenvironment, #10 autophagy, and #11 stress. These clusters delineate the major thematic directions within amino acid deprivation–related oncology research. **Table 6** presents the top burst keywords.

**Table 6:** Keyword emergence related to amino acid deprivation–related cancer research.

Keywords	Year	Strength	Begin	End	2006 - 2025
*in vivo*	2006	31.91	2006	2015	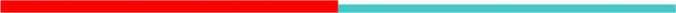
nf kappa b	2006	21.56	2006	2014	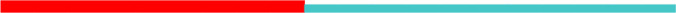
*in vitro*	2006	15.49	2006	2017	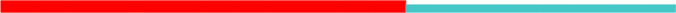
gene	2006	12.79	2006	2013	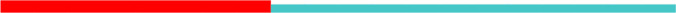
cell cycle	2006	11.92	2006	2013	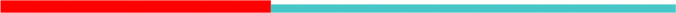
argininosuccinate synthetase	2010	19.62	2010	2018	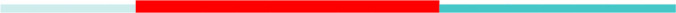
invasion	2010	10.14	2010	2016	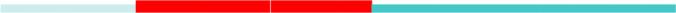
mtor	2014	15.65	2014	2018	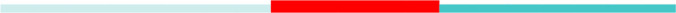
reactive oxygen species	2008	15.58	2015	2019	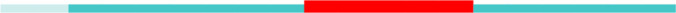
complex	2011	10.73	2015	2018	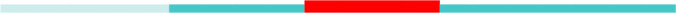
lung cancer	2010	10.44	2016	2018	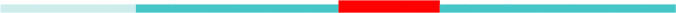
hypoxia	2019	13.88	2019	2025	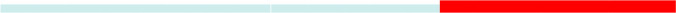
amino acids	2008	11.53	2019	2022	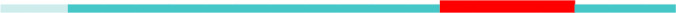
stress	2019	10.21	2019	2025	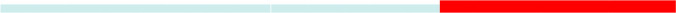
glycolysis	2015	14.51	2020	2023	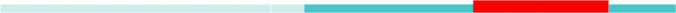
methionine	2020	13.48	2020	2023	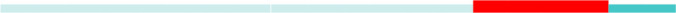
nitric oxide	2008	11.25	2020	2021	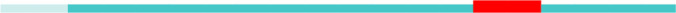
tumor microenvironment	2019	17.13	2021	2025	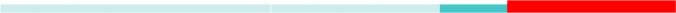
amino acid metabolism	2022	14.74	2022	2025	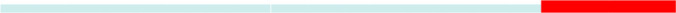
restriction	2017	12.03	2022	2025	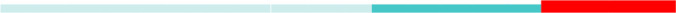
efficacy	2022	11.19	2022	2023	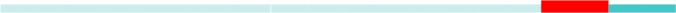
cancer metabolism	2017	10.23	2022	2023	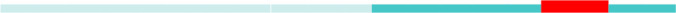
ferroptosis	2020	36.53	2023	2025	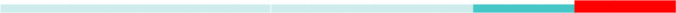
methionine restriction	2019	16.22	2023	2025	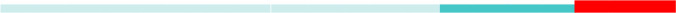
pancreatic cancer	2016	13.89	2023	2025	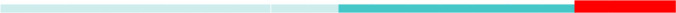

**Figure 6C** presents the keyword time-zone view, where the x-axis indicates the first year a keyword appeared, allowing the research evolution to be summarized into four phases. 2006–2009 was dominated by foundational concepts linking amino acid metabolism (particularly glutamine metabolism) with redox imbalance and cell death. 2010–2014 shifted toward molecular targets and regulatory pathways, highlighted by enzyme vulnerabilities and the emergence of growth-control signaling such as mTOR. 2015–2019 featured more defined intervention strategies alongside methodological advances such as metabolomics. 2020–2025 showed strong expansion and convergence toward translation, with diversification of regulated cell-death programs, increasing attention to specific amino acid pathways, and a clearer shift toward clinically oriented themes including combination therapy, immunotherapy, prognosis, and efficacy.

### 3.6 Comparative analysis of keywords in the PubMed

**Figure 7:**
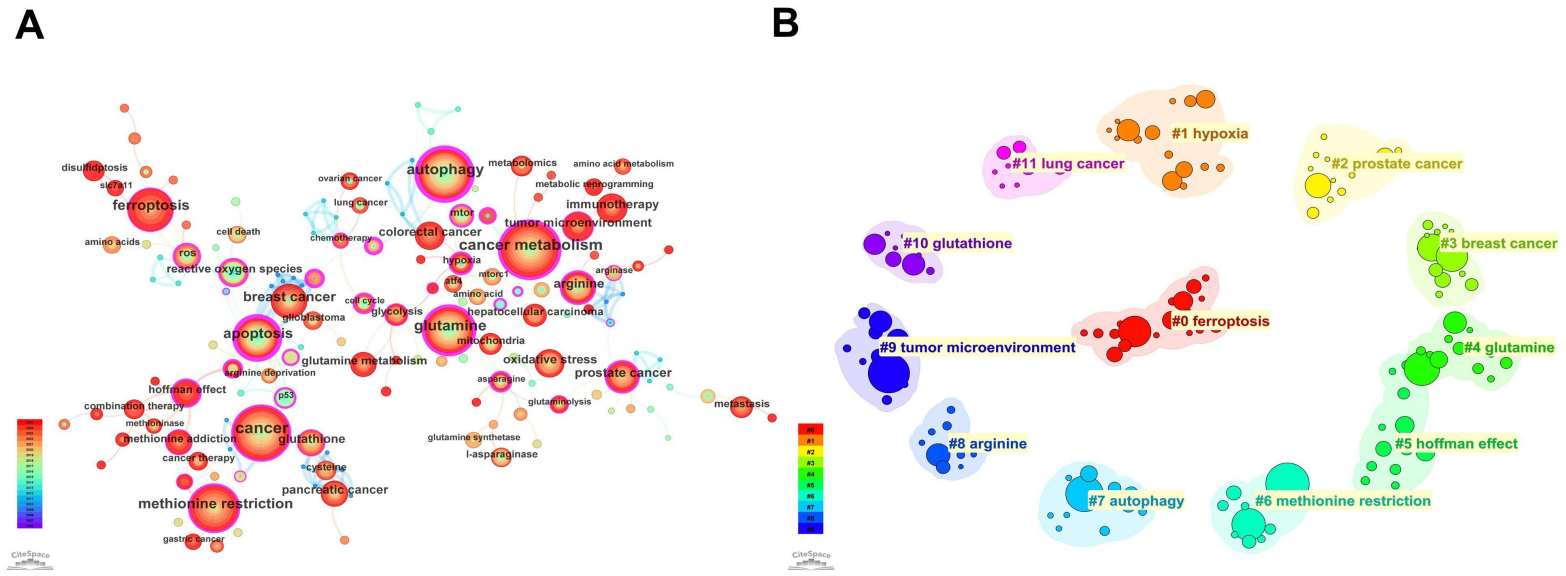
Keyword validation and refinement using PubMed. **(A)** Keyword co-occurrence network derived from the PubMed dataset. **(B)** Keyword clustering map (LLR algorithm) based on PubMed records, used to validate and complement WoSCC-derived themes.

**Figure 7A** presents the keyword co-occurrence network. A side-by-side comparison of PubMed and WoScc co-occurrence patterns indicates that both databases consistently anchor the field around autophagy and apoptosis as core mechanistic themes, with glutamine representing a central metabolic focus. In the PubMed dataset, however, methionine restriction appeared with conspicuously high frequency, accompanied by the prominent emergence of terms such as ferroptosis, cysteine, and SLC7A11, supporting the view that methionine-related interventions and ferroptosis-associated metabolic circuitry currently constitute highly active research frontiers. Moreover, PubMed-specific terms reflecting therapeutic implementation, such as L-asparaginase, arginine deiminase, and recombinant methioninase, together with frequent mentions of chemotherapy, immunotherapy, combination therapy, and drug resistance, suggest that clinically oriented literature places stronger emphasis on concrete intervention strategies, combinatorial regimens, and translational barriers.

**Figure 7B** shows keyword clustering derived from **Figure 7A**. Cluster labels were generated from the keyword field using CiteSpace’s log-likelihood ratio (LLR) algorithm, producing 12 clusters with strong quality metrics (Q = 0.8693, indicating a well-defined modular structure; S = 0.9477, indicating high clustering reliability). The clusters were: #0 ferroptosis, #1 hypoxia, #2 prostate cancer, #3 breast cancer, #4 glutamine, #5 hoffman effect, #6 methionine restriction, #7 autophagy, #8 arginine, #9 tumor microenvironment, #10 glutathione, and #11 lung cancer. Comparison of PubMed and WoS clustering suggests a shared “core map” dominated by methionine dependence–related themes, arginine deprivation, glutamine-centered metabolism, and autophagy-associated signaling, underscoring the stability of foundational research directions. At the same time, disease-oriented clusters and redox-related modules point to precision approaches that exploit tumor-specific metabolic liabilities. Overall, PubMed clustering not only corroborates the principal structure observed in WoScc but also more clearly reflects an ongoing shift toward clinically actionable strategies, emerging therapeutic modalities, and integrative translational research.

### 3.7 Citation and co-citation analysis

**Figure 8:**
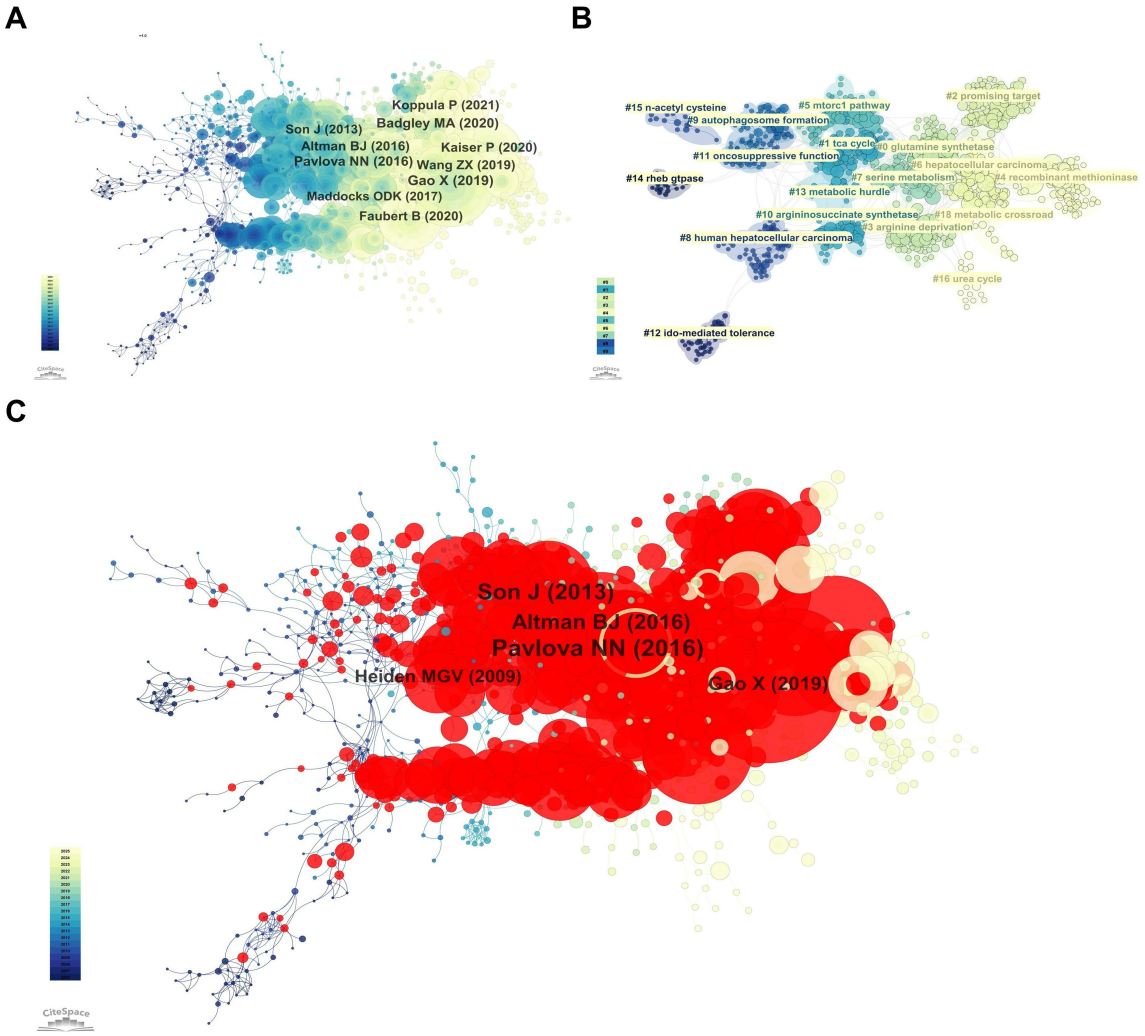
Knowledge base and emerging frontiers from reference analysis. **(A)** Reference co-citation network with labels for highly co-cited references. **(B)** Reference clustering network generated using the LLR algorithm. **(C)** Reference burst detection map highlighting references with strong citation bursts over time.

**Figure 8A** shows the reference co-citation network, with labels marking the 10 most frequently co-cited references (first author and publication year). Among them, the largest node corresponds to the Nature article by Gao, Xia et al. (2019), Dietary methionine influences therapy in mouse cancer models and alters human metabolism (113 co-citations), indicating that it is the most influential and widely acknowledged reference in this field. This study is frequently regarded as a milestone translational work because it provided rigorous evidence that restricting a specific essential amino acid, methionine, can suppress tumor growth across multiple preclinical models by modulating one-carbon metabolism, and can further enhance the efficacy of standard therapies such as 5-fluorouracil–based chemotherapy and radiotherapy. Importantly, the authors also reported early human data suggesting that comparable systemic metabolic shifts can be achieved in humans, thereby advancing the “diet–metabolism–cancer therapy” concept from mechanistic rationale toward a strategy with tangible translational potential. **Table 7** shows the top 25 references by emergence value.

**Table 7:** Bursting references related to amino acid deprivation–related cancer research.

References	Year	Strength	Begin	End	2006 - 2025
Yoon CY, 2007, INT J CANCER, V120, P897, DOI 10.1002/ijc.22322, DOI	2007	20.05	2008	2012	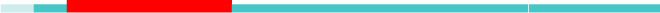
Cheng PNM, 2007, CANCER RES, V67, P309, DOI 10.1158/0008-5472.CAN-06-1945, DOI	2007	19.44	2008	2012	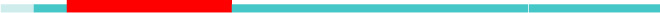
Heiden MGV, 2009, SCIENCE, V324, P1029, DOI 10.1126/science.1160809, DOI	2009	27.22	2010	2014	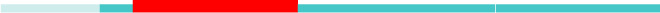
Kim RH, 2009, CANCER RES, V69, P700, DOI 10.1158/0008-5472.CAN-08-3157, DOI	2009	24.95	2010	2014	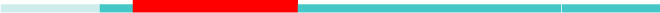
Hanahan D, 2011, CELL, V144, P646, DOI 10.1016/j.cell.2011.02.013, DOI	2011	38.31	2012	2016	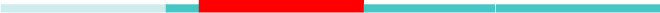
Metallo CM, 2012, NATURE, V481, P380, DOI 10.1038/nature10602, DOI	2012	22.86	2012	2017	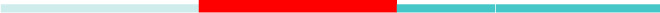
Wise DR, 2010, TRENDS BIOCHEM SCI, V35, P427, DOI 10.1016/j.tibs.2010.05.003, DOI	2010	21.92	2012	2015	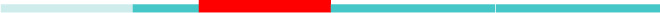
Laplante M, 2012, CELL, V149, P274, DOI 10.1016/j.cell.2012.03.017, DOI	2012	24.69	2013	2017	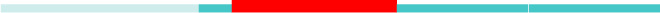
Cairns RA, 2011, NAT REV CANCER, V11, P85, DOI 10.1038/nrc2981, DOI	2011	19.55	2013	2016	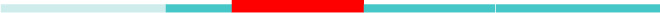
Son J, 2013, NATURE, V496, P101, DOI 10.1038/nature12040, DOI	2013	34.17	2014	2018	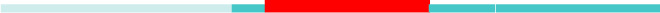
Le A, 2012, CELL METAB, V15, P110, DOI 10.1016/j.cmet.2011.12.009, DOI	2012	23.75	2014	2017	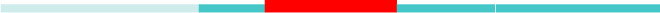
Maddocks ODK, 2013, NATURE, V493, P542, DOI 10.1038/nature11743, DOI	2013	21.35	2014	2018	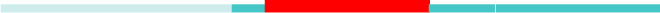
Ward PS, 2012, CANCER CELL, V21, P297, DOI 10.1016/j.ccr.2012.02.014, DOI	2012	20.27	2014	2017	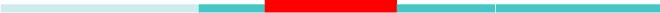
Hensley CT, 2013, J CLIN INVEST, V123, P3678, DOI 10.1172/JCI69600, DOI	2013	25.06	2015	2018	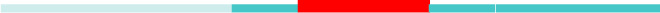
Pavlova NN, 2016, CELL METAB, V23, P27, DOI 10.1016/j.cmet.2015.12.006, DOI	2016	36.14	2017	2021	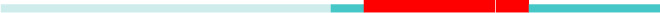
Altman BJ, 2016, NAT REV CANCER, V16, P619, DOI 10.1038/nrc.2016.71, DOI	2016	32.76	2017	2021	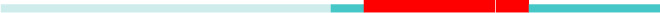
Maddocks ODK, 2017, NATURE, V544, P372, DOI 10.1038/nature22056, DOI	2017	24	2018	2022	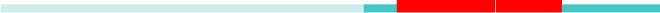
Vander Heiden MG, 2017, CELL, V168, P0, DOI 10.1016/j.cell.2016.12.039, DOI	2017	20.5	2018	2022	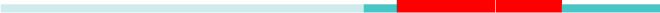
Saxton RA, 2017, CELL, V168, P960, DOI 10.1016/j.cell.2017.02.004, DOI	2017	19.81	2018	2022	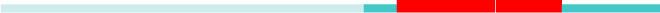
Gao X, 2019, NATURE, V572, P397, DOI 10.1038/s41586-019-1437-3, DOI	2019	29.01	2020	2025	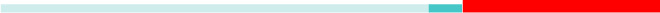
Wang ZX, 2019, NAT MED, V25, P825, DOI 10.1038/s41591-019-0423-5, DOI	2019	21.25	2020	2025	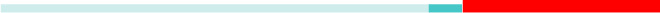
Badgley MA, 2020, SCIENCE, V368, P85, DOI 10.1126/science.aaw9872, DOI	2020	21.74	2021	2025	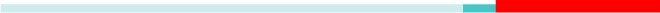
Faubert B, 2020, SCIENCE, V368, P152, DOI 10.1126/science.aaw5473, DOI	2020	23.92	2022	2025	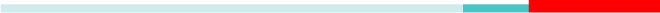
Sung H, 2021, CA-CANCER J CLIN, V71, P209, DOI 10.3322/caac.21660, DOI	2021	25.09	2023	2025	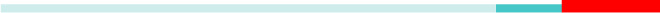
Koppula P, 2021, PROTEIN CELL, V12, P599, DOI 10.1007/s13238-020-00789-5, DOI	2021	21.84	2023	2025	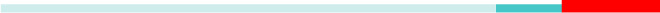

On this basis, a co-citation clustering analysis was performed. Using the LLR algorithm with labels extracted from the title field, 18 major clusters were identified from 1,448 cited references. The resulting co-citation cluster network is presented in **Figure 8B**.

**Figure 8C** highlights burst references within the co-citation network (red nodes), with labels indicating references exhibiting strong burst strength. The strongest burst was observed for Pavlova, Natalya N. et al. (2016) in Cell Metabolism, entitled The Emerging Hallmarks of Cancer Metabolism. Its prominence likely reflects its timely synthesis of a unifying conceptual framework for cancer metabolism, which aligned with—and helped catalyze—a broader shift from single-pathway descriptions toward integrated, systems-level analyses. By explicitly connecting nutrient acquisition (including amino acids such as glutamine) to tumor–microenvironment interactions, immune regulation, epigenetic remodeling, and adaptive strategies under nutrient limitation, this work provided a widely cited foundation for subsequent studies on amino acid deprivation, metabolic plasticity and resistance, and metabolism–immunotherapy combinations, thereby serving as a key intellectual anchor during the field’s rapid expansion.

## 4 Discussion

This study provides a panoramic view of amino acid deprivation–related cancer research and highlights how the field has progressively shifted from foundational metabolic observations to increasingly targetable vulnerabilities and clinically oriented strategies. Overall, the knowledge structure indicates that amino acid targeting is no longer considered an isolated metabolic manipulation, but rather a systems-level intervention that intersects with redox homeostasis, nutrient-sensing signaling, cell fate control, and therapy responsiveness. The sustained growth of publications over the past two decades is consistent with the broader recognition that metabolic reprogramming is integral to tumor progression and treatment resistance, and that amino acid metabolism offers multiple actionable nodes for intervention.

A prominent and persistent theme is the central role of glutamine-centered metabolism. Glutamine functions as a carbon and nitrogen source, supports anaplerosis and nucleotide synthesis, and contributes to antioxidant defense through glutathione-related pathways (18, 19). Its strong network centrality suggests that it acts as a “connector” topic linking oxidative stress, mitochondrial function, and regulated cell death. In parallel, arginine-related themes point to a more vulnerability-driven translational logic: tumors with defects in enzymes such as ASS1 can become auxotrophic and therefore susceptible to arginine depletion strategies (20). This is reflected by the high centrality of argininosuccinate synthetase and by cancer-type–anchored keywords that indicate where metabolic liabilities have been most concretely translated into disease-specific interventions (21). These lines of evidence support a conceptual dichotomy in the field: glutamine research often anchors broad mechanistic understanding, whereas arginine-focused work frequently exemplifies precision targeting based on defined metabolic defects.

Beyond glutamine and arginine, asparagine represents another important amino acid axis that deserves attention in amino acid deprivation-based cancer therapy. Although asparagine-related terms were included in our search strategy, our original discussion did not sufficiently emphasize this emerging theme. Accumulating evidence indicates that asparagine is not merely a substrate for protein synthesis, but also a context-dependent regulator of tumor adaptation, metastatic progression, and therapeutic response. Knott et al. demonstrated that asparagine bioavailability governs metastatic potential in a breast cancer model, suggesting that dietary or enzymatic reduction of asparagine availability may suppress tumor dissemination (22). Mechanistically, Pavlova et al. showed that when extracellular glutamine becomes limited, asparagine becomes essential for maintaining tumor cell survival and proliferation; importantly, asparaginase impaired the ability of cells to adapt to glutamine deprivation, highlighting the metabolic interdependence between glutamine and asparagine pathways (23). In addition, Pathria et al. reported that cancer cells can adapt to asparagine restriction through translational reprogramming and activation of receptor tyrosine kinase-MAPK signaling, indicating that asparagine deprivation can induce compensatory survival programs that may contribute to therapeutic resistance (24). More recently, Chang et al. provided clinically relevant evidence that L-asparaginase-mediated asparagine deprivation may enhance immune-checkpoint blockade by strengthening CD8^+^ T-cell fitness through ROS-mediated metabolic and signaling adaptations (25). Together, these findings suggest that asparagine metabolism links nutrient stress, metastatic competence, adaptive translation, and antitumor immunity.

A second major insight is the field’s diversification of cell-death programs linked to amino acid perturbation. Classical apoptosis and autophagy remain core mechanisms, consistent with the idea that nutrient stress activates conserved survival–death decision circuits (26, 27). However, newer regulated cell death paradigms have increasingly entered the field. Notably, ferroptosis has grown into a substantial research cluster yet remains relatively peripheral in network centrality, suggesting that it is expanding rapidly but has not fully integrated with the main trunk of amino acid deprivation research. This pattern is plausible: ferroptosis research often develops its own conceptual ecosystem centered on lipid peroxidation, cysteine availability, and SLC7A11-driven redox buffering (28, 29). As these links mature, particularly through studies connecting amino acid restriction to redox catastrophe and immune-modulatory consequences, ferroptosis-related work may become more structurally embedded in the broader metabolic-therapy network.

Cross-database comparison further clarifies the translational gradient of the field. PubMed, which often captures more clinically proximate literature and therapeutic framing, shows stronger emphasis on concrete intervention tools and on clinically oriented concepts such as combination therapy, immunotherapy, drug resistance, and efficacy. This contrast is informative rather than contradictory: it suggests that the field has developed two complementary “research languages.” One is mechanistic, focusing on pathways, stress responses, and signaling; the other is intervention-centered, emphasizing deployable modalities and clinical integration. The convergence of these languages is likely to define future progress, particularly in designing rational combinations, selecting responsive populations, and preventing adaptive escape.

The prominence of highly co-cited landmark references also reflects a maturation toward translation. Diet-based methionine restriction studies have become cornerstone citations because they demonstrate, in a clinically meaningful way, that modulating systemic nutrient availability can reshape tumor metabolism and sensitize tumors to standard therapies, with evidence extending beyond preclinical models (30). Such work effectively bridges nutrition, metabolism, and oncology and provides a methodological template for future trials. Likewise, broad conceptual frameworks that define cancer metabolism hallmarks have acted as intellectual accelerators by legitimizing integrated models that incorporate the tumor microenvironment, immune suppression, epigenetic remodeling, and opportunistic nutrient acquisition (31, 32). These frameworks help explain why resistance mechanisms, such as macropinocytosis, metabolic plasticity, and pathway compensation, have emerged as sustained concerns in amino acid deprivation research.

Several translational implications follow. First, therapeutic success will likely depend on patient stratification based on metabolic dependencies rather than on tumor histology alone. Enzyme-deficiency states, transporter reliance, and pathway dominance offer plausible biomarkers, but their clinical utility requires standardized assays and prospective validation (33, 34). Second, amino acid targeting is unlikely to be sufficient as monotherapy in many solid tumors; instead, rational combinations should be prioritized (35). Mechanistically, amino acid restriction can increase oxidative stress and impair repair capacity, providing a rationale for synergy with chemotherapy or radiotherapy. It may also reshape antigen presentation, immune-cell metabolism, or myeloid suppressive programs, supporting combination strategies with immune checkpoint blockade—an area that appears to be emerging but not yet fully integrated into the field core network. Third, resistance and tolerability remain major barriers. Tumors can rewire metabolic fluxes, upregulate compensatory transporters, or engage scavenging pathways, while systemic amino acid restriction may impose nutritional burdens on patients (36). Thus, next-generation strategies may emphasize intermittent scheduling, tumor-targeted delivery, or localized depletion to widen the therapeutic window.

## 5 Conclusion

In summary, amino acid deprivation–related oncology research has evolved into a dynamic and increasingly translational field, anchored by glutamine-centered metabolic networks, vulnerability-driven arginine strategies, expanding cell-death paradigms such as ferroptosis, and growing emphasis on combination regimens and clinical implementation. Future advances will likely depend on deeper integration of mechanistic insight with patient stratification, rational combinations that pre-empt metabolic escape, and clinically feasible intervention platforms that balance efficacy with nutritional safety.

## Data Availability

The original contributions presented in the study are included in the article/supplementary material. Further inquiries can be directed to the corresponding authors.
